# Thromboembolic risks of recombinant factor VIIa Use in warfarin-associated intracranial hemorrhage: a case–control study

**DOI:** 10.1186/1471-2377-12-158

**Published:** 2012-12-15

**Authors:** Chou Sherry H-Y, Cai Xuemei, Konigsberg Rachael G, Bresette Linda M, Henderson Galen V, Sorond Farzaneh A, Feske Steven K

**Affiliations:** 1 Department of Neurology, Brigham and Women’s Hospital, Boston, MA, USA; 2 Harvard Medical School, Boston, MA, USA

**Keywords:** Activated recombinant factor VII, Intracerebral hemorrhage, Thromboembolism, Warfarin

## Abstract

**Background:**

Recombinant factor VIIa (rFVIIa) may be used for rapid hemostasis in life-threatening hemorrhage. In warfarin-associated intracerebral hemorrhage (wICH), FVIIa use is controversial and may carry significant thromboembolic risks. We compared incidence of baseline thromboembolic risk factors and thromboembolism rates in wICH patients treated with additional rFVIIa to those treated with standard therapy of fresh frozen plasma (FFP) and vitamin K alone.

**Methods:**

We identified 45 consecutive wICH patients treated with additional rFVIIa over 5-year period, and 34 consecutive wICH patients treated with standard therapy alone as comparison group. We compared the incidence of post-hemorrhage cardiac and extra-cardiac thromboembolic complications between two treatment groups, and used logistic regression to adjust for significant confounders such as baseline thromboembolic risk factors. We performed secondary analysis comparing the quantity of FFP transfused between two treatment cohorts.

**Results:**

Both rFVIIa-treated and standard therapy-treated wICH patients had a high prevalence of pre-existing thromboembolic diseases including atrial fibrillation (73% vs 68%), deep venous thrombosis (DVT) or pulmonary embolism (PE) (22% vs 18%), coronary artery disease (CAD) (38% vs 32%), and abnormal electrocardiogram (EKG) (78% vs 85%). Troponin elevation following wICH was prevalent in both groups (47% vs 41%). Clinically significant myocardial infarction (MI), defined as troponin > 1.0 ng/dL, occurred in 13% of rFVIIa-treated and 6% of standard therapy-treated patients (p=0.52). Past history of CAD (p=0.0061) and baseline abnormal EKG (p=0.02) were independently associated with clinically significant MI following wICH while rFVIIa use was not. The incidences of DVT/PE (2% vs 9%; p=0.18) and ischemic stroke (2% vs 0%; p=0.38) were similar between two treatment groups. Recombinant FVIIa-treated patients had lower mean INR at 3 (p=0.0001) and 6 hours (p<0.0001) and received fewer units of FFP transfusion (3 vs 5; p=0.003).

**Conclusions:**

Pre-existing thromboembolic risk factors as well as post-hemorrhage troponin elevation are prevalent in wICH patients. Clinically significant MI occurs in up to 13% of wICH patients. rFVIIa use was not associated with increased incidence of clinically significant MI or other venous or arterial thromboembolic events in this wICH cohort.

## Background

Warfarin-associated intracranial hemorrhage (wICH) is growing in incidence and remains the most lethal iatrogenic stroke with a case fatality rate of over 50% [1,2]. The optimal urgent treatment for wICH remains controversial [3]. Available therapies for rapid coagulopathy reversal include intravenous (IV) vitamin K, fresh frozen plasma (FFP), prothrombin complex concentrate (PCC), and rFVIIa [4-12]. Coagulopathy correction with FFP alone takes over 30 hours and 2–4 liters of intravascular volume administration in wICH [7,13]. Recombinant FVIIa is able to bind to tissue factor and increase thrombin generation and platelet activation [14] at the site of hemorrhage; it is considered a possible therapeutic adjunct in life-threatening hemorrhages such as wICH and hemorrhage associated with newer oral anticoagulants.

Intracranial hemorrhage is associated with increased risk of arterial and venous thromboembolic events [15,16]. Though FFP and vitamin K use in wICH do not appear to increase post-ICH thromboembolic complications [17], it is not known whether rFVIIa use in wICH can cause excessive thromboembolic events. In non-anticoagulated ICH patients, the Factor Seven for Acute Hemorrhagic Stroke (FAST) Trial showed rFVIIa use is associated with a small increase in arterial thromboembolism, usually minor cardiac events [18]. Unlike the population studied in FAST, ICH patients on warfarin have pre-existing thromboembolic diseases and may be at much higher risk for subsequent thromboembolic complications as compared to non-anticoagulated ICH patients. In this study, we compare the baseline thromboembolic risk factors and the incidence of cardiac and extra-cardiac thromboembolic complications in two groups of wICH patients, one treated with standard therapy consisting of FFP transfusion and IV vitamin K, and the other treated with standard therapy plus additional 40 μg/kg of rFVIIa.

## Methods

### Patient selection

We identified 80 consecutive rFVIIa-treated ICH patients admitted to a single tertiary care hospital from 2003–2008 from a prospective registry. We excluded 30 patients who received anticoagulants other than warfarin and 5 who had multi-system trauma and secondary disseminated intravascular coagulation, leaving 45 patients for analysis. For comparison, we identified a cohort of 34 consecutive patients with wICH who were treated with IV vitamin K and FFP alone. To minimize patient selection bias, we selected this comparison cohort from the time period of 2008–2009 when rFVIIa was no longer available at our institution as adjunct therapy to wICH. This study was approved by the local institutional review committee (Partners Human Research Committee) and procedures followed were in accordance with institutional guidelines.

### Thromboembolism definition

We systematically reviewed all patient records for baseline thromboembolic risk factors and pre-defined cardiac and extra-cardiac thromboembolic complications. We defined event-related troponin elevation as any troponin-I elevation above normal laboratory assay range within 72 hours of rFVIIa or FFP administration. We defined clinically-significant MI as elevation of troponin-I above 1.0 ng/dL with or without EKG changes within 72 hours of rFVIIa or FFP administration.

Since EKG changes following ICH are common and can be mediated by the primary neurological event, we systematically analyzed all EKGs using pre-defined definition for new EKG changes to minimize interpretation bias. Two reviewers (SC and XC) reviewed all EKGs and classified them under the following categories: i) ST segment changes, ii) T-wave changes, iii) conduction block, iv) any arrhythmia, and v) QTc prolongation.

We defined DVT as the presence of thrombi in the deep venous system detected by duplex ultrasound or computed tomography (CT) venogram anytime during acute hospitalization for wICH. PE was defined as the presence of thrombi on chest CT angiography. Duplex venous ultrasound, CT venography, and chest CT angiography were performed according to standardized clinical protocol based on clinical suspicions and not as routine screening. We defined new ischemic strokes as the presence of new lesions on head CT or MRI consistent with ischemic infarction anytime during acute hospitalization for wICH. All wICH patients underwent head CT or MRI upon initial presentation and again at 24 hours following wICH presentation per standardized clinical protocol. Additional head CT or MRI was acquired based on clinical indications. Peripheral arterial ischemia was defined as clinical exam findings consistent with limb ischemia not present on admission.

We systematically reviewed patient records for baseline thromboembolic risk factors, including prior coronary artery disease (CAD), atrial fibrillation (AF), ischemic stroke, transient ischemic attack (TIA), DVT, PE, and history of malignancy. Patients were considered to have a history of CAD if they carried a diagnosis of angina, MI, ischemic cardiomyopathy, or had undergone coronary artery angioplasty, stenting, or cardiac bypass surgery.

### Clinical and outcome data

We collected data on patient demographics, indications for warfarin therapy, treatment of wICH, in-hospital mortality, and INR and partial thromboplastin time (PTT) at hospital presentation and at 3 and 6 hours following rFVIIa or FFP infusion, and total FFP use throughout hospital stay. For patients who died prior to hospital discharge, we recorded the causes of death and changes in goals of care.

### Standardized wICH treatment protocol

All patients with ICH were treated with a standardized protocol including reduction of systolic blood pressure to below 160 mmHg and infusion of IV vitamin K and FFP if the INR was above 1.4. FFP infusion continued until there was no clinical evidence of continued bleeding and INR returned ≤ 1.4. All patients were screened for cardiac ischemia which included 3 sets of EKG and cardiac troponin-I measurements at 8 hour intervals. In patients with troponin elevation or EKG changes, troponin-I and EKG were repeated daily until results stabilized. All patients with suspected DVT or PE underwent duplex ultrasound, CT venography, or chest CT angiography. All patients received thromboembolism prophylaxis with compression stockings, sequential compression devices, and subcutaneous heparinoid injections.

From 2003–2008, rFVIIa was available at our institution for compassionate use in wICH. Patients with INR ≥ 1.5 and acute ICH who did not have acute ischemic EKG changes or troponin-I elevation were eligible to receive rFVIIa 40 μg/kg at the physician’s discretion.

### Statistical analysis

We compared all continuous variables using two-sided Student *t*-test for normally distributed and Wilcoxon rank sum test for non-normally distributed data. Categorical variables were compared using the chi-square test. We used multivariate logistic regression models to explore the independent relationship between troponin elevation and clinically significant MI with rFVIIa and with other risk factors. Age, history of cancer, and baseline abnormal EKG were included as covariates because they were significantly different between the two cohorts. Additional factors were selected as covariates if they were associated with the outcome of interest (troponin elevation, clinically significant MI) on univariate screening with significance of p < 0.1. All statistical analyses were performed using JMP 8.0.

## Results

### Pre-existing thromboembolic conditions

Baseline characteristics of the rFVIIa-treated and comparison cohorts of wICH patients were similar (Table 1). Both cohorts consisted of elderly patients (73–77 years) with predominantly intra-parenchymal hemorrhage (IPH) or subdural hemorrhage (SDH). The most common indication for warfarin therapy was AF, followed by DVT/PE.

**Table 1:** Baseline Characteristics

**Characteristics**	**FVIIa Group (n = 45)**	**Comparison Group (n = 34)**	**p-value**
Age	73 [69–76]	77 [74–81]	0.07
Female gender	22 (49%)	19 (56%)	0.53
Diagnosis			0.77
Intra-parenchymal hemorrhage	22 (49%)	14 (41%)	
Subdural hemorrhage	19 (42%)	17 (50%)	
Subarachnoid hemorrhage	4 (9%)	3 (9%)	
Indication for anticoagulation			0.79
AF	29 (64%)	21 (62%)	
Mechanical valve	3 (7%)	2 (6%)	
DVT/PE	7 (16%)	5 (15%)	
Stroke	2 (4%)	2 (6%)	
Cardiomyopathy	1 (2%)	3 (9%)	
Unknown	3 (7%)	1 (3%)	
Prior Thromboembolism History			
History of AF	33 (73%)	23 (68%)	0.58
History of CAD	17 (38%)	11 (32%)	0.62
History of DVT/PE	10 (22%)	6 (18%)	0.61
History of stroke/TIA	8 (18%)	10 (29%)	0.22
History of cancer	5 (11%)	11 (32%)	0.02
Abnormal baseline EKG	35 (78%)	29 (85%)	0.01
T wave abnormality	11 (24%)	2 (6%)	
ST abnormality	11 (22%)	12 (35%)	
Conduction block	5 (11%)	7 (21%)	
Arrhythmia	6 (13%)	6 (18%)	
QTc prolongation	1 (2%)	2 (6%)	
Left ventricular hypertrophy	2 (4%)	0	
Initial INR	2.47 [2.17 – 2.78]	2.22 [1.98 – 2.47]	0.36
Initial PTT	40.3 [35.8 – 44.8]	36.8 [34.2 – 39.4]	0.75

Both groups had similarly high prevalence of pre-morbid AF (73% in rFVIIa-treated group vs 68% in comparison group), CAD (38% vs 32%), DVT/PE (22% vs 18%), and stroke/TIA (18% vs 29%). The standard-therapy wICH cohort had more subjects with history of malignancy or an abnormal baseline EKG. Prevalence of ST segment abnormalities on baseline EKG was similar in both groups. No one had ST segment elevation on baseline EKG.

### Cardiac thromboembolic complications

Over 93% of study cohort had troponin-I data, and all subjects with EKG changes had concurrent measurement of cardiac troponin-I.

Troponin elevation following wICH was prevalent (Table 2), and the rates of troponin elevation were similar between two study cohorts. Troponin elevations were rarely accompanied with concurrent EKG changes. This occurred in two patients in rFVIIa-treated group and none in the comparison group (4% v.s. 0%; p = 0.29). The EKG changes observed here were T-wave inversion and QTc prolongation thought to be secondary to the primary neurological event.

**Table 2:** Incidences of Thromboembolic Complications

	**FVIIa Group (n = 45)**	**Comparison Group (n = 34)**	**p-value**
Troponin elevation	21 (47%)	14 (41%)	0.53
Clinically Significant Troponin Elevation (Troponin > 1.0 ng/dL)	6 (13%)	2 (6%)	0.52
New EKG changes	19 (42%)	6 (18%)	0.06
T-wave abnormality	8 (18%)	1 (3%)	
ST abnormality	4 (9%)	3 (9%)	
Conduction block	1 (2%)	0	
Arrhythmia	3 (7%)	1 (3%)	
QTc prolongation	2 (5%)	1 (3%)	
Troponin elevation with EKG Changes	2 (4%)	0	0.29
DVT/PE	1 (2%)	3 (9%)	0.18
Stroke	1 (2%)	0	0.38

New EKG changes occurred in 42% of rFVIIa-treated cohort and 18% of standard-therapy wICH cohort, with the predominant EKG changes being T-wave abnormalities. There were no cases of ST elevation or ventricular arrhythmias in either cohort. The incidence of clinically significant troponin elevation (troponin-I > 1.0 ng/dL) in rFVIIa-treated cohort was 13% compared to 6% the standard-therapy cohort (p = 0.52). Only one patient with troponin-I elevation had hemodynamic instability, which occurred transiently during propofol infusion.

We explored the relationship between clinically-significant MI in wICH and baseline thromboembolic risk factors or rFVIIa use. Thromboembolic risk factors explored included age, gender, history of DVT/PE, history of AF, history of CAD, history of cancer, history of stroke/TIA, and abnormal EKG at baseline. On univariate screening, clinically-significant MI (troponin-I > 1.0 ng/dL) in wICH was associated with prior history of CAD (p=0.0095) and presence of baseline abnormal EKG (p=0.03) but not with rFVIIa use (p=0.46). In the multivariate logistic regression model, past history of CAD (p = 0.0061) and abnormal baseline EKG (p = 0.02) were independently associated with development of clinically significant MI following wICH while rFVIIa use was not (p = 0.81) associated with this outcome.

### Extra-cardiac thromboembolic complications

The incidence of venous thromboembolism (DVT or PE) during acute hospitalization was less prevalent than elevated cardiac biomarkers overall, and the incidences were similar between two patient cohorts (Table 2). No one in either cohort developed peripheral arterial occlusion.

Ischemic cerebral events were rare. One patient from the rFVIIa-treated cohort and none from the standard-therapy cohort developed an ischemic stroke during acute hospitalization. This patient had pre-existing atrial fibrillation, had not received antithrombotic therapy since wICH onset, and developed small ischemic strokes in multiple vascular territories consistent with cerebral emboli 20 days after rFVIIa treatment.

### Clinical outcomes

The rFVIIa-treated cohort had lower INR at 3 and 6 hours following treatment and received fewer units of FFP (Table 3) compared the standard-therapy cohort. More rFVIIa-treated patients underwent surgical hematoma evacuation (38% v.s 18%, p = 0.05) as compared with the standard-therapy cohort. Overall, there is no significant correlation between surgical hematoma evacuation and death (p=0.583). However, in the rFVIIa-treated cohort, there was significantly higher survival with surgical hematoma evacuation compared to the standard-therapy cohort (53.6% v.s 82.4%, p=0.044). This association is not seen in the cohort treated with conventional therapy (86% v.s 67%, p = 0.28).

**Table 3:** Clinical Outcomes

	**FVIIa group (n = 45)**	**Comparison Group (n = 34)**	**p-value**
Mean INR at 3 h	0.98 [0.90 – 1.06]	1.63 [1.40 – 1.86]	0.0001
Mean INR at 6 hours	1.10 [1.00 – 1.20]	1.51 [1.41 – 1.61]	<0.0001
Mean units of FFP transfused	3 [2.1 – 3.9]	5 [3.6 – 6.3]	0.003
Surgical hematoma evacuation	17 (38%)	6 (18%)	0.05
Survival after surgical hematoma evacuation	14 (31%)	4 (12%)	0.04
In-hospital death	16 (36%)	6 (18%)	0.07
Withdrawal of life-prolonging care	14 (31%)	6 (18%)	0.17

In-hospital mortality was 36% in the rFVIIa-treated cohort and 18% in the standard-therapy cohort (Table 3). In the rFVIIa-treated cohort, mortality was higher in patients with IPH (55%) and SAH (75%) than in those with SDH (5%). Fourteen of sixteen deaths in the rFVIIa-treated cohort and all six deaths in the standard-therapy cohort occurred following withdrawal of life-sustaining therapy because of poor neurologic prognosis. The remaining two patients met criteria for brain death. No patient died as a result of thromboembolic complications.

## Discussion

In this study, we found wICH patients have a high prevalence of baseline thromboembolic risk factors and high incidence of post-ICH troponin elevation regardless of rFVIIa exposure. The overall incidence of clinically-significant MI (troponin-I > 1.0 ng/dL) ranged from 6-13% and was not significantly different between rFVIIa-treated compared to standard-therapy wICH cohorts. Recombinant FVIIa use also did not appear to increase the incidence of post-ICH extra-cardiac thromboembolic events in our cohorts. In addition, thromboembolic complications did not contribute to any in-hospital deaths in either wICH treatment group; rather, all deaths were attributable to the primary neurological disease. While a prior history of CAD and abnormal EKG at baseline may confer a higher risk of clinically significant MI following wICH, rFVIIa use did not appear to increase the risk of post-ICH troponin elevation or clinically significant MI after adjusting for risk factors.

Compared with the Factor Seven for Acute Hemorrhagic Stroke (FAST) study [14], we found a higher incidence of troponin elevation (41-47%) regardless of rFVIIa use in these wICH patient cohorts. This difference likely arose from the fact that FAST included non-anticoagulated ICH patients whose baseline risk for thromboembolism may be very different from wICH patients. As we found in this study, wICH patients tend to have a high prevalence of baseline risk factors for thromboembolism. The FAST cohort had an average age of 65, 12% baseline thromboembolism history, and only 5% history of prior MI. In comparison, our wICH patients were older (mean age 73–77 years) and had a much higher prevalence of prior thromboembolic diseases, including a 32-38% prevalence of pre-existing CAD. FAST found that rFVIIa use in non-anticoagulated ICH patients was associated with a dose-dependent increase in the combined incidence of non-ST elevation MI and ST-elevation MI (STEMI) (6.4% in placebo group – 12.1% in 80μg/kg FVIIa group). Despite higher prevalence of baseline risk factors in our wICH cohort, we did not observe any STEMI in this patient cohort.

Clinical outcome analysis suggest that the rFVIIa treated cohort had lower INR at 3 and 6 hours, which is a known effect of rFVIIa on INR measurements and may not reflect reversal of warfarin-associated coagulopathy. The fact that rFVIIa treated cohort also received fewer units of FFP transfusion and were more likely to undergo surgical evacuation compared to conventional therapy cohort may be secondary to their lower INR measurements, or to clinical evidence of hemostasis. While surgical evacuation is not associated with overall mortality, we observe an interesting association between surgical evacuation and higher rate of survival in the rFVIIa-treated cohort but not in the cohort treated by conventional therapy. This association may suggest that rFVIIa-treated cohort derived more benefit from surgical hematoma evacuation while the conventional-therapy cohort did not. A prospective, randomized study would be necessary to this postulated association between rFVIIa treatment and potential benefit from surgical hematoma evacuation.

To our knowledge, this is the largest study comparing thromboembolic complications of rFVIIa use to FFP and IV vitamin K therapy in warfarin-related ICH. Compared to a large case series by Robinson and colleagues, where 101 patients treated with variable doses of rFVIIa for warfarin-associated hemorrhages of the brain and spinal canal had no cardiac thromboembolic events [19], we found a much higher incidence of cardiac thromboembolic events following wICH regardless of rFVIIa use. Differences in location of hemorrhages, methods use for cardiac thromboembolism screening, and differences in rFVIIa dosage likely have contributed to the difference in results between our study and the study by Robinson et al. Our results on rFVIIa-related extra-cardiac thromboembolism are more consistent with that reported by Robison et al. (DVT 10%, ischemic stroke 3%) and by the FAST study (DVT 4-5%, PE 1%, ischemic stroke 3-6%). These results suggest a relatively lower overall incidence of extra-cardiac thromboembolic complications in ICH regardless of warfarin or rFVIIa use.

Consistent with prior studies [20], the rFVIIa-treated cohort in this study achieved lower mean INR values than the standard-therapy wICH group. This effect on INR was sustained for at least 6 hours following rFVIIa administration, and the rFVIIa-treated cohort received less FFP. While recombinant FVIIa is known to normalize INR rapidly in warfarin-associated systemic bleeding [21], whether such INR normalization reflects full reversal of coagulopathy remains controversial [22-24] though several prior studies of rFVIIa use in wICH did report potential clinical therapeutic benefits [20,23,25-28]. Because this study was not designed to measure the therapeutic effect rFVIIa in hemostasis in wICH, we must interpret these data with caution. The fact that rFVIIa-treated group required less FFP may reflect that rFVIIa-treated patients had clinical evidence of hemostasis with less FFP use, but a prospective study would be necessary to determine whether rFVIIa promotes improve outcome in wICH.

This study has several limitations. Although one of the larger studies of rFVIIa use in wICH, our overall sample size is small and this limits our power to detect small effects. The observed rate of 13% *versus* 6% clinically significant MI may reflect a true difference in cardiac ischemia risk associated with rFVIIa use which may be detected in a larger cohort. The retrospective design subjects this study to potential biases such as selection and data ascertainment bias. To minimize patient-selection bias, we chose a comparison standard-therapy wICH cohort from a time period when rFVIIa was not available on formulary. This avoids the most serious systematic errors in selecting patients with lower thromboembolic risks for rFVIIa therapy. Existence of a standardized clinical protocol for cardiac and extra-cardiac thromboembolism screening in all wICH patients limits the potential for data ascertainment bias in this particular study. This study does not address the comparative thromboemboilc complication rates and efficacy of warfarin reversal of rFVIIa compared to other potential agents such as PCC. Though we have established the prevalence of thromboembolic complications from rFVIIa use in wICH, the thromboembolic risks of PCC use in this population is still unknown. While rFVIIa as an activated factor capable of direct binding to tissue factor suggest it may achieve faster hemostasis, PCC contains all vitamin-K dependent coagulation factors and may be a more efficacious agent for warfarin reversal. Further studies are necessary to address both of these important questions. Finally, we chose to analyze all patients with intra-cerebral hemorrhage as a single cohort in this study, as has been done in other studies of rFVIIa use in wICH [19,23,28]. Though this design increases generalizability of our data on systemic thromboembolic effects of rFVIIa use in intracranial hemorrhages in general, it introduces heterogeneity in the neurological conditions and their subsequent treatments, thereby limiting our ability to study the specific effects of rFVIIa on neurologic outcomes of wICH. Despite these limitations, this study is the first to report the overall incidence of cardiac and extra-cardiac thromboembolic complications with rFVIIa use in wICH, and to provide a comparison with thromboembolic risks of wICH patients treated with standard therapy.

## Conclusions

Warfarin-associated ICH patients have high prevalence of baseline thromboembolic risk factors and high prevalence of post-ICH troponin elevation regardless of rFVIIa use. Recombinant FVIIa use does not appear to increase the risk of cardiac or extra-cardiac thromboembolic events following wICH in our patient cohorts. Pre-existing CAD and abnormal baseline EKG are risk factors for clinically significant MI post-wICH regardless of rFVIIa use. Recombinant factor VIIa is not associated with prohibitively high incidence of thromboembolic complications while it is associated with decreased FFP use and higher survival rate after hematoma evacuation, making it a potential wICH therapeutic agent. Larger prospective randomized studies are necessary to determine whether rFVIIa or PCC use improves outcome in wICH patients.

## Abbreviations

AF: Atrial fibrillation; CAD: Coronary artery disease; DVT: Deep venous thrombosis; EKG: Electrocardiogram; FAST: Factor Seven for Acute Hemorrhagic Stroke study; FFP: Fresh frozen plasma; IPH: Intra-parenchymal hemorrhage; PCC: Prothrombin complex concentrate; PE: Pulmonary embolism; rFVIIa: Recombinant factor VIIa; SAH: Subarachnoid hemorrhage; STEMI: ST-elevation myocardial infarction; SDH: Subdural hemorrhage; TIA: Transient ischemic attack; wICH: Warfarin-associated intracerebral hemorrhage.

## Competing interests

SH-Y Chou received funding support from the American Heart Association (10CRP2610341), and the Harvard CTSC/NIH (5KL2RR025757-02), and NIH/NINDS (5K23NS073806).

## Authors’ contributions

SC: Conceived and designed study, collected data, performed the statistical analysis, and prepared manuscript. XC: Identified study subjects, acquired and interpreted data, performed statistical analysis, and prepared manuscript. RK and LB: Identified study subjects, collected data, assisted in manuscript preparation. GH, FS, and SF: acquired and interpreted data, prepared manuscript. All authors read and approved the final manuscript.

## Pre-publication history

The pre-publication history for this paper can be accessed here:

http://www.biomedcentral.com/1471-2377/12/158/prepub
